# Rapid and inexpensive bedside diagnosis of RAN binding protein 2-associated acute necrotizing encephalopathy

**DOI:** 10.3389/fneur.2023.1282059

**Published:** 2023-11-16

**Authors:** Benoît Gouy, Adrien Decorsière, Sophie Desgraupes, Wenming Duan, Hong Ouyang, Yifan E. Wang, E. Ann Yeh, Alexander F. Palazzo, Theo J. Moraes, Sébastien Nisole, Nathalie J. Arhel

**Affiliations:** ^1^Institut de Recherche en Infectiologie de Montpellier, University of Montpellier, Montpellier, France; ^2^Master de Biologie, École Normale Supérieure de Lyon, Université Claude Bernard Lyon 1, Université de Lyon, Lyon, France; ^3^Program in Translational Medicine, The Hospital for Sick Children Research Institute, Toronto, ON, Canada; ^4^Department of Biochemistry, University of Toronto, Toronto, ON, Canada; ^5^Department of Pediatrics, Division of Neurology, The Hospital for Sick Children, University of Toronto, Toronto, ON, Canada; ^6^Division of Neuroscience and Mental Health, The Hospital for Sick Children Research Institute, University of Toronto, Toronto, ON, Canada; ^7^Department of Pediatrics, Division of Respiratory Medicine, The Hospital for Sick Children, Toronto, ON, Canada

**Keywords:** acute necrotizing encephalopathy, RANBP2, nuclear pore complex, RGPD, diagnostic test, screening tools, biomarker, neuropediatric

## Abstract

Acute necrotizing encephalopathy 1 (ANE1) is a very rare disorder associated with a dominant heterozygous mutation in the *RANBP2* (RAN binding protein 2) gene. ANE1 is frequently triggered by a febrile infection and characterized by serious and irreversible neurological damage. Although only a few hundred cases have been reported, mutations in *RANBP2* are only partially penetrant and can occur *de novo*, suggesting that their frequency may be higher in some populations. Genetic diagnosis is a lengthy process, potentially delaying definitive diagnosis. We therefore developed a rapid bedside qPCR-based tool for early diagnosis and screening of ANE1 mutations. Primers were designed to specifically assess *RANBP2* and not *RGPD* (RANBP2 and GCC2 protein domains) and discriminate between wild-type or mutant *RANBP2*. Nasal epithelial cells were obtained from two individuals with known *RANBP2* mutations and two healthy control individuals. *RANBP2*-specific reverse transcription followed by allele-specific primer qPCR amplification confirmed the specific detection of heterozygously expressed mutant *RANBP2* in the ANE1 samples. This study demonstrates that allele-specific qPCR can be used as a rapid and inexpensive diagnostic tool for ANE1 using preexisting equipment at local hospitals. It can also be used to screen non-hospitalized family members and at risk-population to better establish the frequency of non-ANE-associated *RANBP2* mutations, as well as possible tissue-dependent expression patterns.

**Systematic review registration:**

The protocol was registered in the international prospective register of systematic reviews (PROSPERO– CRD42023443257).

## 1 Introduction

Familial acute necrotizing encephalopathy 1 (ANE1) is a rare disorder associated with a dominant heterozygous mutation in the *RANBP2* gene (RAN binding protein 2) (1, 2). Magnetic resonance imaging (MRI) findings include bilateral and symmetric T2 hyperintensities of the thalamus and pons, with frequent hemorrhagic transformation. Neurological involvement typically follows an infectious prodrome. It is characterized by high rates of morbidity, with mortality in up to 30% in some series (1). Early recognition and treatment with anti-inflammatory therapies, such as steroids, IVIG, and plasmapheresis may result in improved outcomes (3–5). Genetic diagnosis typically takes weeks, potentially delaying definitive diagnosis. Rapid bedside testing may therefore be of benefit.

**Figure 1:**
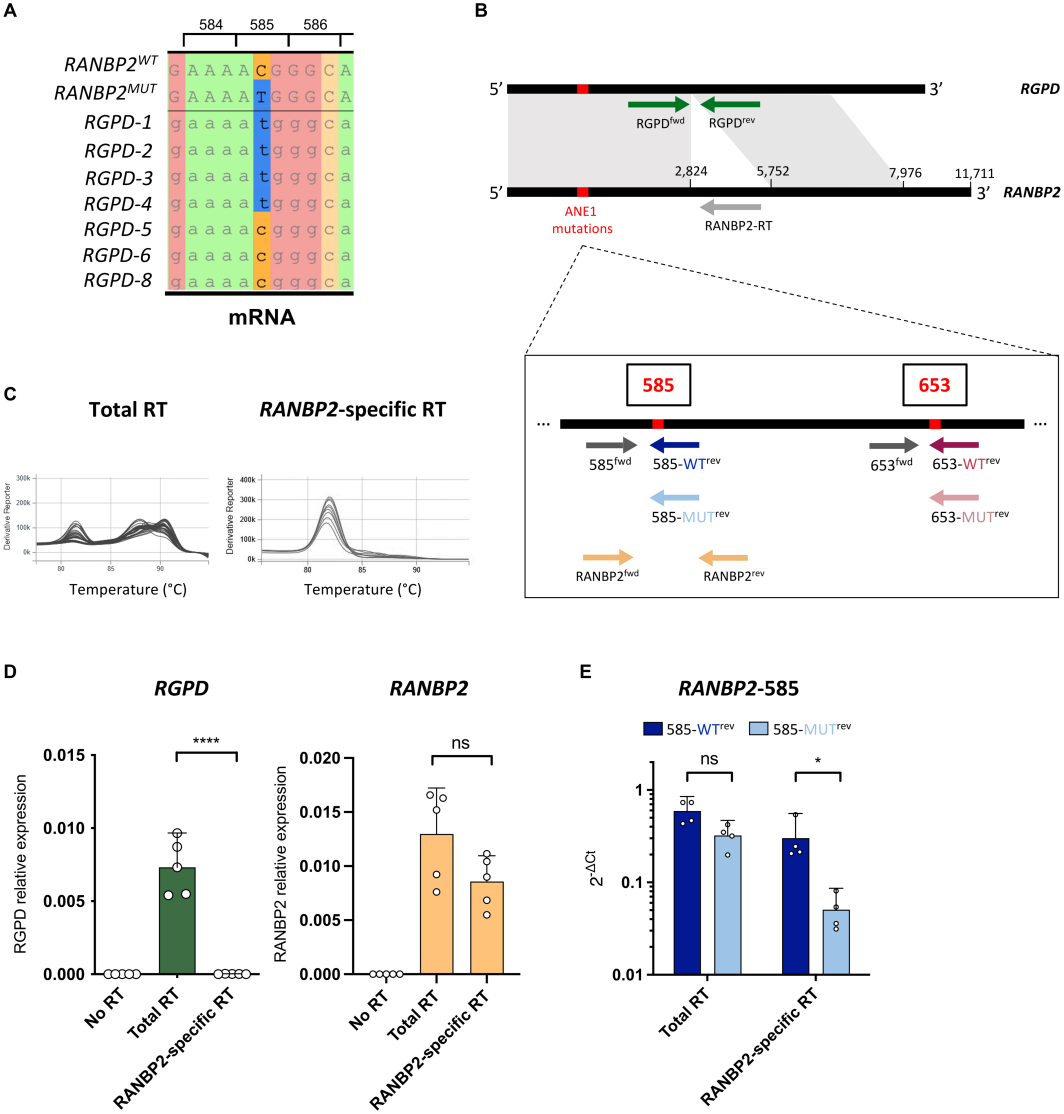
*RANBP2*-specific RT eliminates the detection of *RGPD*. **(A)** Local alignment of *RANBP2*, *RANBP2*-*T585M*, and *RGPD* mRNAs illustrate the presence of the c.1880C>T genotype in 4 of the 7 *RGPDs*. Codon positions correspond to *RANBP2*. **(B)** Position of the primers that were used for *RANBP2*-specific RT and allele-specific qPCR of wild-type and mutant *RANBP2*. Common sequences between *RANBP2* and *RGPD* are indicated in grey. **(C–E)** RNAs were extracted from THP-1 cells, and used for *RANBP2*-specific RT (with *RANBP2*-RT primer), total RT (with poly dT and random primers), or no RT (without reverse transcriptase). **(C)** Representative melting curves using RANBP2^fwd^/RANBP2^rev^ primers show the amplification of multiple different transcripts with total RT and of a probable single transcript with *RANBP2*-specific RT. **(D)** qPCR was performed using RGPD^fwd^/RGPD^rev^ (left) or RANBP2^fwd^/RANBP2^rev^ (right) sets of primers. $C_{t}$ values were normalized against the mean of *RPL13a* from the total RT. Results show 5 independent experiments and each dot corresponds to the mean of qPCR triplicates. Error bars represent a 95% confidence interval. **(E)** qPCR was performed using allele-specific primers on total RT or *RANBP2*-specific RT. $C_{t}$ values were normalized against the mean of total *RANBP2*. Results show 4 independent experiments and each dot corresponds to the mean of qPCR triplicates. For all graphs, statistical significance was determined using paired *t*-test (two-tailed), ns, not significant, ^*^p ≤ 0.05 and ^****^p ≤ 0.0001.

The predominant ANE1 variant involves a single point mutation c.1880C>T that translates into a T585M substitution in the RANBP2 protein (2, 6). Other variants have also been described, such as c.2085C>T (T653I), and all lead to missense mutations that are clustered in the 5′ end of the coding sequence. However, this region is highly conserved in seven *RGPD* (RANBP2 and GCC2 protein domains) genes that resulted from duplication and recombination of *RANBP2* (7–9) and 4 out of 7 *RGPDs* also harbor the c.1880C>T genotype (Figure 1A), thus confounding the detection of ANE1 variants by classical qPCR approach.

Here, we developed a qPCR-based diagnostic tool that allows the rapid detection of the predominant *RANBP2* mutations in patients and can be deployed to screen affected families or at-risk populations. To exclude *RANBP2* paralogs, we designed primers to first specifically amplify *RANBP2* using reverse transcription PCR (RT-PCR), and then performed allele-specific qPCR to identify point mutations. The method is a cost-effective (<10 €/patient) and rapid (time to result <1 day) bedside diagnostic tool for the genetic susceptibility to ANE1 that only requires routine qPCR equipment present in most hospital services.

## 2 Methods

### 2.1 Patient samples/consent and ethic

Nasal epithelial cells were obtained from two individuals with known RANBP2 mutations (T585M) and two healthy control individuals after obtaining informed consent (REB#1000061106, The Hospital for Sick Children, Toronto). For this work, related specifically to RANBP2 (secondary use REB#1000071481) a total of 4 donor cells were studied; 2 participants were healthy controls and reported to be free from known lung disease or ANE1; 2 participants were family members with known abnormalities in the *RANBP2* gene, and had MRI and/or clinical abnormalities consistent with those seen in acute necrotizing encephalopathy of childhood (ANEC) (5). Clinical and demographic features are provided in Table 1. Cells were expanded in submerged culture (Pneumacult Ex, StemCell Tech, Vancouver, Canada) and differentiated at air liquid interface (Pneumacult ALI, StemCell Technologies) as previously described (10). RNA was extracted from cells after 28 days of ALI culture.

**Table 1:** Patient demographic and clinical features.

	ID	Age	Sex	Clinical features
Patient	460	11 yrs	F	Optic atrophy, developmental delay, seizures
	461	46 yrs	F	No clinical symptoms
Control	456	6 yrs	F	
	387	41 yrs	F	

F, female; yrs, years.

### 2.2 Sequence alignment

RANBP2 and RGPD mRNA sequences from Ensembl data base (ENST00000283195.11, ENST00000398193.8, ENST00000398146.5, ENST00000304514.11, ENST00000408999.4, ENST00000016946.8, ENST00000329516.8, ENST00000302558.8), were aligned on CLUSTAL website and Jalview.

### 2.3 Cell lines and culture

HEK293T and THP-1 cells were grown in Dulbecco’s Modified Eagle Medium (DMEM, Gibco) and Roswell Park Memorial Institute medium (RPMI, Gibco), respectively. Culture media were complemented with 10% of inactivated FCS (Gibco), and 1% Penicillin/Streptomycin (Gibco). They were maintained in culture at 37°C in 5% CO_2_.

### 2.4 Plasmids and transfections

Scramble shRNA was a gift from David Sabatini (Addgene plasmid #1864). We purchased the RANBP2 shRNA plasmid from Sigma (TRCN0000003454) and modified it by replacing the *puromycin resistance* gene with an *mCherry-puromycin resistance* fusion gene that was amplified from pCDH-CMV-mCherry-T2A-Puro (a gift from Kazhuhiro Oka, Addgene plasmid #72264), and inserted using restriction-enzyme cloning.

The plasmids pEGFP-C2-RANBP2, pEGFP-BPN (1-993aa) and pEGFP-BPN-T653I were kindly provided by Jomon Joseph (National Centre for Cell Science, Pune, India). The pEGFP-C2-RANBP2-T585M mutant was generated by site-directed mutagenesis using the QuickChange Site-Directed mutagenesis kit (Agilent).

Plasmids were transfected into HEK293T cells using calcium phosphate precipitation and cells were harvested 48 h post-transfection.

### 2.5 Sorting FACS

Cells were trypsinized, washed in PBS, pelleted, and resuspended at 5 × 10^6^ cell/mL in PBS, 5 nM EDTA (Invitrogen), 2% FCS. mCherry-positive cells were sorted on a FACSAria IIu (Becton Dickinson) and recovered in PBS-50% FCS prior to RNA extraction.

### 2.6 RNA extraction and reverse-transcription

Total RNA was extracted using RNeasy Mini kit (Qiagen) and treated with DNase (RNase-Free DNase Set, Qiagen), following the manufacturer’s instructions. RNA concentration and purity were evaluated by spectrophotometry (Implen NanoPhotometer N60). Up to 500 ng of RNA were reverse transcribed using the PrimeScript RT Reagent Kit (Perfect RealTime, Takara Bio Inc.) following the manufacturer’s instructions. Two types of reaction were performed in order to reverse transcribe either all transcripts (Total-RT) or only RANBP2 transcripts (RANBP2-RT). Total-RT reactions were performed at 37°C using both oligo dT and random primers, whereas RANBP2-RT were performed at 55°C with the RANBP2-RT primer (5′- ATGCTGTTGGGGTGGAAGCC- 3′). Control reactions without RT were also performed.

### 2.7 Quantitative PCR

Real-time PCR reactions were performed in triplicate using Takyon ROX SYBR MasterMix blue dTTP (Eurogentec) on an Applied Biosystems QuantStudio 5 (Thermo Fisher Scientific) in 384-well plates. Transcripts were quantified using the following program: 3 min at 95°C followed by 35 cycles of 15 s at 95°C, 20 s at 60°C, and 20 s at 72°C, min, followed by a melting curve acquisition step. All primers are listed in Table 2.

**Table 2:** Sequence of primers used to quantify transcripts by qPCR.

Name	Sequence (point mutations are indicated in bold)
RPL13A^fwd^	AACAGCTCATGAGGCTACGG
RPL13A^rev^	TGGGTCTTGAGGACCTCTGT
RGPD^fwd^	ACTCCCACAAAGGGTTCTTCTAA
RGPD^rev^	TCCTGGTTCCGAAATGCCAA
RANBP2^fwd^	AAAACATGGCCTTCAACCTG
RANBP2^rev^	TCAACAATTTCTGATGCCTGA
585^fwd^	TGGGATGCGGTTTGTACTCT
585-WT^rev^	AGAATTAAGACCGCTGCCC**G**
585-MUT^rev^	AGAATTAAGACCGCTGCCC**A**
653^fwd^	AGTGTAGACATTCAGGCATCAGA
653-WT^rev^	CCATTTACTGCATCCAATATAGCAAAA**G**
653-MUT^rev^	CCATTTACTGCATCCAATATAGCAAAA**A**

^*^RGPD primers amplify all 7 RGPD coding sequences.

Values for each transcript were normalized to the expression levels of *RPL13A* (60S ribosomal protein L13a) in the conditions of Total-RT. To compare amplifications of *RANBP2* using point-mutation-specific primers, $C_{t}$ values were normalized to the $C_{t}$ values of total RANBP2 from RANBP2-specific RT.

### 2.8 PCR and sequencing

To verify patient heterozygosity of *RANBP2* transcripts at position 585, the region flanking this position was amplified by PCR from donor or patient cDNA, using the primers 585^fwd^ and RanBP2^rev^. Amplicons were submitted to Sanger Sequencing (Eurofins Genomics).

### 2.9 Statistics

Statistical analyses were performed using the version is 9.5.1 of GraphPad PRISM.

## 3 Results

To specifically assess *RANBP2* and not *RGPDs*, the 5′ region of *RANBP2* was first reverse transcribed (RT) using a *RANBP2*-specific primer (RANBP2-RT) on human THP-1 cells (Figure 1B). We then examined qPCR amplifications of *RGPD* and *RANBP2* on *RANBP2*-specific RT samples compared with classical RT using poly dT and random primers, and found that the *RANBP2*-specific RT successfully eliminated the detection of *RGPD* transcripts without impacting the detection of *RANBP2* (Figures 1C,D). Of note, because of the presence of *RANBP2* paralogs in humans (9), the sequencing of *RANBP2* would also need to be performed using RNA and a *RANBP2*-specific primer for first-strand synthesis (1). However, in contrast to previous work which positioned the primer binding site in the 3′ untranslated region of *RANBP2* (1), we designed the RANBP2-RT primer to bind to the closest region to the 5′ end of the coding sequence to increase the efficiency and accuracy of the RT.

To quantify the allelic expression of ANE1-associated risk variants, we developed a qPCR assay that compares the relative expression of WT and mutant *RANBP2* transcripts in human samples. For this purpose, we designed allele-specific primers with a single 3′ base variation to discriminate between WT and mutant *RANBP2* (Figure 1B) (11, 12). Using primer sets to detect C or T nucleotides at the coding sequence position 1880 (protein position 585), we confirmed that WT and mutant *RANBP2* could not be discriminated after total RT (Figure 1E), because of the concomitant detection of the wild-type C nucleotide in *RGPD5-8* and the mutant T nucleotide in *RGPD1-4* in all samples (Figure 1A). However, using *RANBP2*-specific RT ensured the detection of wild-type RANBP2 with only minimal detection using mutant-specific primers, suggesting good allele-specific discrimination (Figure 1E).

**Figure 2:**
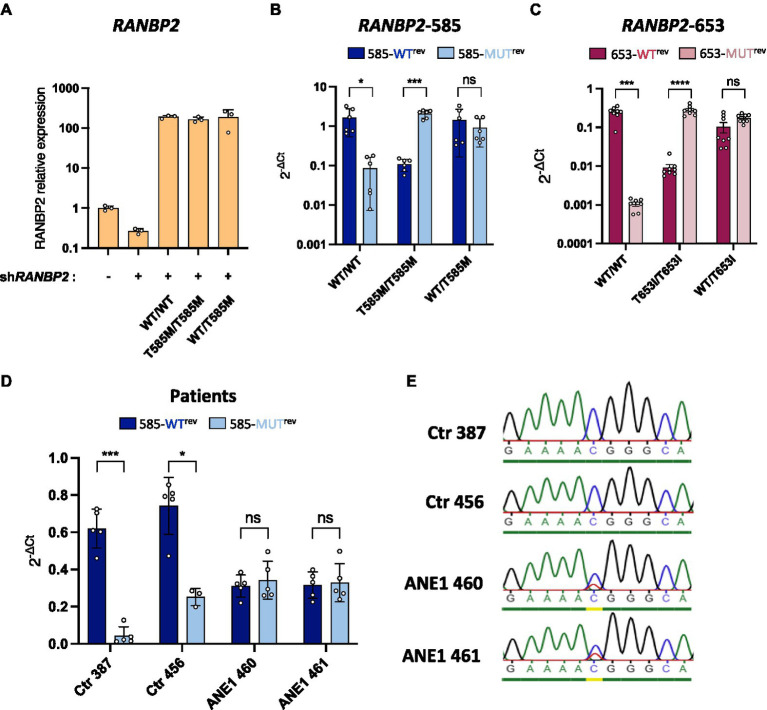
Allele-specific qPCR discriminates between WT and mutant *RANBP2*. **(A–C)** HEK293T cells were co-transfected with a *RANBP2*-targeting shRNA to knockdown endogenous expression and plasmids expressing either wild-type *RANBP2*, mutant *RANBP2*, or both. As a control, cells were transfected with a control shRNA or left untransfected. Cells were sorted by FACS (BD Aria) prior to RNA extraction. **(A)** qPCR amplification of total *RANBP2* shows the degree of knockdown achieved and the equivalent expression levels of the WT and T585M RANBP2 constructs. The graph shows values normalized for *RPL13a* from a representative experiment performed in triplicate. **(B,C)** Allele-specific-qPCR detection of mutations at protein position 585 **(B)** or 653 **(C)** were performed and normalized against the amplification of total *RANBP2* using RANBP2^fwd^/RANBP2^rev^ primers $\left(2^{-\Delta C_{t}}\right)$ . Values are the means from 2 independent experiments performed in triplicate ± SD. **(B)** Allele-specific qPCR was performed using primer 585^fwd^ with either 585-WT^rev^ or 585-MUT^rev^. **(C)** Allele-specific qPCR was performed using primer 653^fwd^ with either 653-WT^rev^ or 653-MUT^rev^. **(D)** RNA extracts from ANE1 subjects and healthy control subjects (Ctr) were used for *RANBP2*-specific RT. qPCR was performed using RANBP2^fwd^/RANBP2^rev^ primers (total *RANBP2*), or allele-specific 585^fwd^ with either 585-WT^rev^ or 585-MUT^rev^ primers. $C_{t}$ values were normalized against the mean of total *RANBP2*. Values are the means from 2 independent experiments performed in triplicate ± SD. **(E)** PCR was performed on RT products from *F*, using 585^fwd^ and RANBP2-RT primers. PCR products were purified on gel and sequenced. For all graphs, statistical significance was determined using paired *t*-test (two-tailed), ns, not significant, ^*^p ≤ 0.05, ^***^p ≤ 0.001, and ^****^p ≤ 0.0001.

Next, we applied *RANBP2*-specific RT and qPCR to diagnose ANE1 mutants in an experimental cell model. HEK293T cells were depleted of endogenous RANBP2 and transfected to express WT or ANE1 variants either hetero- or homozygously using equimolar transfection ratios (Figure 2A). We found that the WT-specific primers amplified the WT cDNA at a similar efficiency relative to the RANBP2-total primers, and weakly amplified the 585-MUT or 653-MUT cDNA (ratio of 0.001–0.1). Reciprocal results were obtained with the mutation-specific primers, while heterozygous expression was amplified equally by both sets of primers (Figures 2B,C). These results indicate that the method can robustly distinguish heterozygotes from homozygotes at ANE1-predisposition loci on *RANBP2*.

To demonstrate that this approach can be used to diagnose individuals, we performed allele-specific qPCR on nasal epithelial cells obtained from ANE1 subjects and healthy control subjects. Genetic testing can in theory be performed on any tissue, including on blood samples. However, nasal testing is less invasive and therefore can be rapidly and easily performed in patients at the bedside, including in young children in whom blood draws may be challenging. Moreover, nasal testing makes the collection of DNA from multiple individuals quite easy, allowing for the screening of cohorts of non-hospitalized individuals. Mutation-negative individuals (Ctr) showed predominant detection by WT-specific primers, whereas ANE1 subjects had a 50/50 amplification profile, indicating heterozygous expression of the mutation at position 585 (Figure 2D). The phenotype was confirmed by Sanger sequencing (Figure 2E), indicating a complete agreement in the allelic assignments made qPCR assay and classical sequencing.

## 4 Discussion

In this pilot study, we evaluated whether qPCR can be used to successfully identify ANE1-susceptibility *RANBP2* mutants, using both *in vitro* models of homozygous/heterozygous expression, and nasal swabs from 4 individuals. Results indicated that the method can distinguish heterozygotes from homozygotes at ANE1-predisposition loci on *RANBP2*, and distinguish between ANE1 patients and control individuals. We therefore succeeded in developing a system that discriminates WT and mutant alleles of *RANBP2*, despite the existence of the locus of interest in multiple other *RGPD* genes.

The method provides a cost-effective alternative to sequencing that can be applied to diagnose genetic susceptibility to ANE1 rapidly, to determine the relative expression of alleles in different tissues, or to screen at-risk populations. Specifically, the costs for genetic screening using classical PCR amplification and sequencing of exon 14 alone are estimated at 160 €/patient (in France), whereas the total cost of allele-specific qPCR is under 10 €/patient. It should also be noted that, because of the presence of *RANBP2* paralogs in humans, the detection of ANE1 mutations using sequencing requires the same initial procedure of RNA extraction and *RANBP2*-specific first strand synthesis as our approach, whose cost is ~8 €/patient. Therefore, the difference in cost between exon sequencing and allele-specific qPCR is in fact >150 €/patient versus <2 €/patient, respectively. Additionally, while classical genetic diagnosis can take weeks, allele-specific qPCR can be performed on the day of admission, as is routinely performed to sequence SARS-CoV-2 variants for instance.

One limitation is that the technique only identifies already known mutations, however the majority of ANE1-associated mutations are c. 1880C>T; p. Thr585Met (73%) (2), suggesting that screening at position 585 using allele-specific qPCR is a good starting strategy. Alternative allele-specific primers can also quite readily be designed, and we demonstrated the feasibility of the approach using another mutation, c.2085C>T; p.Thr653Ile. It should also be noted that, because of the very large size of *RANBP2*, sequencing of the entire gene consisting of 29 exons is very labor and cost-intensive, and that screening of ANE1 mutations usually involves the sequencing exon 14 only (1), which also precludes the detection of new mutations in other regions of *RANBP2*.

Another limitation is that this pilot study was performed on 4 participants only, two of which had known mutations of the RANBP2 gene and were of the same family. ANE1 is an extremely rare condition and only ~100 individuals have been reported to this day (2). Although this pilot study demonstrates that allele-specific qPCR can be used to diagnose ANE1, the next steps should be to test this in a larger cohort including children with a variety of known abnormalities in the *RANBP2* gene versus a larger control cohort.

In conclusion, we posit that allele-specific qPCR detection of ANE1 mutations could allow genetic screening to be made routinely on hospitalized ANE cases, in a manner that is compatible with the time constraints of clinicians and the equipment available on site. Moreover, the low cost of this approach makes it accessible to services with limited resources, particularly in low and middle-income countries. This rapid and inexpensive screen could be used as a first step, and a decision to send for further testing (whether by sequencing or WES/WGS) could then be made. Additionally, having demonstrated that the allele-specific qPCR diagnosis can be performed on nasal swabs, we illustrate the potential of the approach to screen non-hospitalized individuals, for instance, to estimate the prevalence within a population or to determine the genetic predisposition within families of ANE patients.

## Data availability statement

The original contributions presented in the study are included in the article/supplementary material, further inquiries can be directed to the corresponding author.

## Ethics statement

The studies involving humans were approved by REB#1000061106, The Hospital for Sick Children, Toronto. The studies were conducted in accordance with the local legislation and institutional requirements. Written informed consent for participation in this study was provided by the participants’ legal guardians/next of kin.

## Author contributions

BG: Conceptualization, Data curation, Formal analysis, Investigation, Methodology, Writing – original draft, Writing – review & editing. AD: Conceptualization, Data curation, Investigation, Methodology, Validation, Writing – original draft, Writing – review & editing. SD: Validation, Visualization, Writing – original draft, Writing – review & editing. WD: Resources, Writing – review & editing. HO: Resources, Writing – review & editing. YW: Resources, Writing – review & editing. EY: Resources, Writing – original draft, Writing – review & editing. AP: Resources, Writing – original draft, Writing – review & editing. TM: Resources, Writing – original draft, Writing – review & editing. SN: Conceptualization, Data curation, Formal analysis, Methodology, Validation, Writing – original draft, Writing – review & editing, Investigation. NA: Conceptualization, Funding acquisition, Supervision, Writing – original draft, Writing – review & editing, Resources.
